# Additive value of pre-operative and one-month post-operative lymphocyte count for death-risk stratification in patients with resectable pancreatic cancer: a multicentric study

**DOI:** 10.1186/s12885-016-2860-6

**Published:** 2016-10-26

**Authors:** Christelle d’Engremont, Dewi Vernerey, Anne-Laure Pointet, Gaël Simone, Francine Fein, Bruno Heyd, Stéphane Koch, Lucine Vuitton, Stefano Kim, Marine Jary, Najib Lamfichek, Celia Turco, Zaher Lakkis, Anne Berger, Franck Bonnetain, Julien Taieb, Philippe Bachellier, Christophe Borg

**Affiliations:** 1 Department of Gastroenterology, University Hospital of Besançon, Besançon, France; 2 Methodology and Quality of Life in Oncology Unit, University Hospital of Besançon, Besançon, France; 3 Department of Gastroenterology and GI oncology, Paris Descartes University, Georges Pompidou European Hospital, Paris, France; 4 Department of Digestive Surgery and Liver Transplantation, University hospital of Strasbourg, Strasbourg, France; 5 Department of Digestive Surgery and Liver Transplantation, University Hospital of Besançon, Besançon, France; 6 Department of Medical Oncology, University Hospital of Besançon, Besançon, France; 7 Department of Digestive Surgery, Hospital of Belfort-Montbeliard, Montbeliard, France; 8 Department of GI Surgery, Paris Descartes University, Georges Pompidou European Hospital, Paris, France; 9 Centre investigation Clinique en biothérapie, CIC-1431 Besançon, France; 10 UMR1098 INSERM/Université de Franche Comté/Etablissement Français du Sang, Besançon, France; 11 Department of Oncology, University Hospital of Besançon, 3 Boulevard Alexander Fleming, Besancon, F-25030 France

**Keywords:** Pancreatic adenocarcinoma, Lymphocyte count, Lymphopenia, Prognosis

## Abstract

**Background:**

Pancreatic adenocarcinoma (PDAC) incidence is increasing worldwide. Several studies have shown that lymphopenia was correlated with a poor prognosis but the potential interest to measure lymphopenia in the pre and post-operative setting as well as its added value among conventional prognostic factors was never investigated.

**Methods:**

Data from two independent cohorts in whom patients underwent resection for pancreatic carcinoma were retrospectively recorded. We examined the association between perioperative findings, pre and post-operative lymphocyte counts and overall survival (OS) in univariate and multivariate analyses. Performance assessment and internal validation of the final model were evaluated with Harrell’s C-index, calibration plot and bootstrap sample procedures.

**Results:**

Three hundred ninety patients were included in the analysis between 2000 and 2011. Pre and post-operative lymphocyte counts were independent prognostic factors associated with OS in multivariate analysis (*p* = 0.0128 and *p* = 0.0764, respectively). The addition of lymphocyte count variable to the conventional parameters identified in multivariate analysis (metastatic lymph node ratio, veinous emboli and adjuvant chemotherapy) significantly improved the model discrimination capacity (bootstrap mean difference = 0.04; 95 % CI, 0.01–0.06). The use of a threshold and combining the categorical (≥1000; <1000) information in pre and post lymphocyte counts permitted the identification of 4 subgroups of patients with different prognosis (*p* < 0.0001). Finally, the description of patients in long-term remission showed that only 3 of 65 (4.6 %) patients with post-operative lymphocyte count under 1000/mm^3^ were alive 4 years after surgery contrary to 54 of 236 (22.8 %) patients with a post-operative lymphocyte count above 1000/mm^3^.

**Conclusion:**

Pre and post-operative lymphopenia are independent prognostic factors for OS and they have an additive value regarding conventional prognostic factors for death-risk stratification and to predict long-term survival. Lymphopenia should be included as stratification factors in future clinical trial assessing overall survival in pancreatic cancer patients.

**Electronic supplementary material:**

The online version of this article (doi:10.1186/s12885-016-2860-6) contains supplementary material, which is available to authorized users.

## Background

Pancreatic ductal adenocarcinoma incidence and mortality is increasing worldwide and is one of the leading causes of cancer-related death [1–3]. Surgical pancreatic resection is the only curative treatment. However, patients treated by surgery and adjuvant chemotherapy only achieved 10–20 % of long-term survival [4, 5]. Then, the identification of prognostic factors correlated with the risk of early relapse is an important issue to better optimize neoadjuvant or adjuvant treatments.

Current recognized prognostic factors include mainly pathological parameters such as lymph node invasion, tumour grade and resection margin involvement [6]. However, these conventional clinical parameters as well as gene mutation status are insufficient to predict accurately death-risk and the probability of long-term remission [7, 8].

A growing body of evidence suggests that immune-related biomarkers are correlated with survival in several cancers. A high infiltration of tumours by CD8+ T cell lymphocytes (TILs) was correlated to a good prognosis in colorectal and pancreatic cancers [9–11]. Conversely, cancers endowed with the ability to escape the immune system are expected to display a worse prognosis. Inflammation, recruitment of suppressive immune cells (regulatory T lymphocytes or myeloid derived suppressor cells) or induction of lymphocyte-apoptosis mediated by tumour cells, are potential mechanisms leading to immune escape [12]. Peripheral blood lymphocyte count to assess lymphopenia in cancer patients might be a convenient and clinically relevant option to identify cancers associated with an enhanced risk of tumour immune evasion and poor prognosis.

Several studies have shown that an elevated preoperative neutrophil-to-lymphocyte ratio and a decreased preoperative lymphocyte count allow the prediction of chemotherapy-related toxicities [13, 14]. Moreover, pre-operative lymphopenia might be a prognostic factor for several cancer patients [15–17]. In pancreatic cancer, immune suppression at baseline is also correlated with overall survival. However, despite the number of studies conducted so far, lymphocyte count parameter is not used in current practice, probably because its additive value regarding conventional prognostic markers is still not widely known [18–22]. Furthermore, recent advances in immunology evidenced that chemotherapy might promote an immunogenic cell death leading to an increased anti-tumour immunity [23]. The presence of a lymphopenia after surgery might impact the efficacy of adjuvant chemotherapy. Having observed in current clinical practice that most patients with pre-operative lymphopenia have a lymphocyte count above 1000/mm3 1-month post surgery, we hypothesized that the prognostic value of lymphopenia might be more accurate when assessed after pancreatic adenocarcinoma removal. Consequently, we decided to conduct a study based on two independent cohorts including 390 patients treated by surgery for a localized pancreatic adenocarcinoma. The aims of this study were: i) to conduct a confirmatory study to validate the prognosis value of pre-operative lymphopenia, ii) to assess the impact of post-operative lymphopenia on overall survival, iii) to discriminate the additive value of pre and post-operative lymphocyte counts among conventional prognosis factors.

## Methods

### Population

This retrospective analysis included data from two independent cohorts of patients with histologically confirmed pancreatic adenocarcinoma, who underwent a curative intent surgery. The first cohort included patients treated in a university hospital (Georges Pompidou European Hospital, henceforth referred as HEGP) and in a regional cancer institute (university hospital of Besançon, Belfort and Montbeliard general hospital) between January 1, 2000 and April 30, 2010. The second cohort included patients treated in the university hospital of Strasbourg (Hautepierre) between January 1, 2004 and December 31, 2011. We excluded patients with other histopathological type of cancers: cholangiocarcinoma, neuroendocrine tumor and patients for whom preoperative lymphocyte count parameter was not available. Data from the two cohorts were updated in june, 2013.

### Data collection

The following data were collected at diagnosis for each patient: center and patient identification, age, sex, diabetes, level of albuminemia which is the presence of albumin in blood, pathological characteristics and prescription of adjuvant chemotherapy. Lymphocyte counts on routine blood tests were recorded the day before the surgery and 1 month after the surgery. Deaths were collected during the follow-up of the study for each patients. All data from the two cohorts were extracted from the paper files and the desktop folder for each patient. The software was ChimioProd and Dxcare, Millenium, Axigate and Dxcare for HEGP, Belfort and Montbeliard, Besançon and Strasbourg Hospitals respectively. Adjuvant chemotherapy data from Belfort, Montbeliard and Besançon hospitals were excerpted from a regional register (BPC software).

### Statistical analysis

We provided the mean (SD) values and frequency (percentages) for the description of continuous and categorical variables, respectively. The means and the proportion were compared using Student’s t test and the chi-squared test (or Fisher’s exact test, if appropriate), respectively. Due to the skewed lymphocyte count distribution we used for its description the median, and the interquartile range for the dispersion measurement, as recommended [24]. Wilcoxon rank-sum test was performed for lymphocyte count distribution comparison among the cohort set.

Overall Survival (OS) was calculated from the date of surgery to the date of death from any cause. Alive patients were censored at the last follow-up. OS was estimated using Kaplan Meier method and described using median or rate at specific time points with 95 % confidence intervals (CI). Follow-up was calculated using reverse Kaplan-Meier estimation.

The association of parameters at enrolment with OS was assessed using univariate Cox to produce the hazard ratio (HR) and 95 % confidence intervals firstly for pre-operative parameters, tumoral, therapeutic and lymphocyte count factors. Separate multivariate cox-analysis in conventional factors block (pre-operative, tumoral and therapeutic) and lymphocyte count block were performed with stepwise backward – elimination with the inclusion of variable with *p* < 0.05 in univariate analysis. The factors identified in these analyses were thereafter included in a final multivariate model with stepwise backward – elimination. When used in continuous in the Cox modelisation, lymphocyte count variable had to be normalized by logarithmic transformation, considering its skewed distribution. The lymph node ratio was defined by the ratio of the number of involved lymph nodes reported to the total number of lymph nodes removed in the lymph node dissection. The threshold 0.2 was kept as proposed in the study of Yamamoto et al. [25]. Hazard proportionality was checked by plotting log-minus-log survival curves.

Accuracy of the final model was checked regarding two parameters: discrimination and calibration. The predictive value and the discrimination ability of the model were evaluated with Harrell’s Concordance (C)-index. One thousand random samples of the population were used to derive 95 % CI for the Harrell’s Concordance statistic. Calibration and goodness of fit of the model were assessed by using the extension of Hosmer-Lemeshow test and for survival analysis and *p*-value greater than 0.1 was considered as an indicator for acceptable agreement. Calibration was also assessed by visual examination of calibration plot. Internal validity of the model was assessed by a bootstrap sample procedure. Several approaches have been proposed to assess the performance in samples of the same population (internal validation). Sensitivity analyses were performed for univariate and multivariate Cox models with a stratified approach on the cohort set parameter that allowed considering the two cohort heterogeneities in the Cox modelisation.

The predictive value that lymphocytes counts variables added to a reference risk model (all parameters identified in the multivariate final model except lymphocytes counts variables) was evaluated with the use of C-statistic. This analysis was repeated 1000 times using bootstrap samples to derive 95 % CI for the difference in the C-statistics between the two models in order to finally, assess the improvement in discrimination of lymphocytes counts parameters among the other conventional parameters.

We also used net continuous reclassification improvement (NRI) and integrated discrimination improvement (IDI) to quantify the performance and the net benefit of the addition of lymphocyte count to the reference model for the prediction of 48 months death probability. Continuous NRI has several limitations but would give a consistent message and is therefore a descriptive marker. One should note, cNRI does not consider the magnitude of the change, but only the direction. This is done by the IDI. When significantly greater than 0, IDI and cNRI are in favour of a net benefit of the addition of the marker of interest to the reference model considered.

Then, we investigated the possibility to provide a simple implementation of lymphocyte count parameter in clinical practice, guided by the determination for a relevant cut-off in order to categorize patients at baseline and post-operative time. According to the study of *Ray-Coquard and al* the threshold chosen was 1000 cells/mm^3^ [26]. In addition, in clinical current practice considerations, patients with a lymphocyte count lower than 1000 are commonly considered in a lymphopenic status.

Finally, considering the added value of lymphocyte count measurements for OS risk stratification among conventional factors and their independent association with OS, we investigated the interest for a combination of pre and post-operative lymphocyte count in clinical practice.

The analyses were conducted using SAS 9.2 (Statistical Analysis System, Cary, NC, USA) and R 3.0.2 (R foundation for Statistical Computing). All statistical tests were 2-sided, and probability values <0.05 were regarded as significant.

## Results

### Population

Characteristics of the overall population and according to the two cohort sets are given in Table 1. A total of 390 patients with resectable pancreatic cancers were enrolled. There were 192 (49,2 %) patients in cohort 1 and 198 (50,8 %) in cohort 2. Pathological findings differed significantly between the two cohorts but the most frequent T stage was T3-T4 in both cohorts (85 and 94 % respectively in cohort 1 and 2; *p* < 0.01). However OS of the patients in the two cohorts were not significantly different (Additional file 1: Figure S1, HR = 0.870 95 % CI: 0.696–1.088 *p* = 0.2235).

**Table 1:** Baseline characteristics of the overall population and according to the two cohort sets

	Patients Characteristics	Overall population (*N* =390)		Cohort set 1 (*N* =192)		Cohort set 2 (*N* =198)		*P*
		*N*		*N*		*N*		
Pre-operative parameters	*Age at surgery — years*	390		*192*		*198*		
	*≤ 65*		174 (45 %)		*87 (45 %)*		*87 (44 %)*	
	*> 65*		216 (55 %)		*105 (55 %)*		*111 (56 %)*	0.8387
	Patient male sex *— no. (%)*	390	222 (57 %)	192	103 (54 %)	198	119 (60 %)	0.2201
	Diabetes *— no. (%)*	325		186		139		
	No		252 (78 %)		143 (77 %)		109 (78 %)	
	Yes		73 (22 %)		43 (23 %)		30 (22 %)	0.7891
	Albuminemia g/L	191	36.4 ± 7.7	70	32.6 ± 6.4	121	38.6 ± 7.4	<0.0001
	Tumor Size in cm	301	3.8 ± 1.6	164	3.6 ± 1.5	137	4.0 ± 1.6	0.0095
Tumoral post-operative parameters	pT Local invasion *— no. (%)*	375		184		193		
	0-1-2		39 (10 %)		28 (15 %)		11 (6 %)	
	3–4		336 (90 %)		154 (85 %)		182 (94 %)	0.0022
	N status	388		190		198		
	0		119 (31 %)		68 (36 %)		51 (26 %)	
	1		269 (69 %)		122 (64 %)		147 (74 %)	0.0365
	Positive lymph nodes ratio	380	0.15 ± 0.19	182	0.20 ± 0.23	198	0.12 ± 0.14	<0.0001
	Lymph Nodes ratio							
	< 0.2	380	279 (73 %)		117 (64 %)		162 (81 %)	
	≥ 0.2		104 (27 %)		67 (36 %)		37 (19 %)	<0.0001
	Histological Grade	331		160		171		
	Poorly differentiated		85 (26 %)		23 (14 %)		62 (36 %)	
	Moderately differentiated		158 (48 %)		76 (48 %)		82 (48 %)	
	Well differentiated		88 (26 %)		61 (38 %)		27 (16 %)	<0.0001
	Vascular invasion	361		186		175		
	No		242 (67 %)		115 (62 %)		127 (73 %)	
	Yes		119 (33 %)		71 (38 %)		48 (27 %)	0.0335
	Lymphatic invasion	364		189		175		
	No		230 (63 %)		107 (57 %)		123 (70 %)	
	Yes		134 (37 %)		82 (43 %)		52 (30 %)	0.0089
	Perineural invasion	365		191		174		
	No		138 (38 %)		57 (30 %)		81 (47 %)	
	Yes		227 (62 %)		134 (70 %)		93 (53 %)	0.0012
Therapeutic post-operative parameter	Adjuvant Chemotherapy	336	251 (75 %)	183	118 (64 %)	153	133 (87 %)	<0.0001
Lymphopenia parameters	Pre-operative lymphocyte count^a^	390	1458.9 (150–8350)	194	1492.1 (170–3052)	199	1426 (150–8350)	0.3362
	Post-operative lymphocyte count^a^	299	1410 (200–32000)	127	1400 (251–3620)	172	1430 (200–32000)	0.2657
Follow-up parameters	Death events	393	309 (79 %)	192	157 (82 %)	198	152 (77 %)	0.2233
	Median F-up time in months 95 % CI^b^		66.6 (60.8–78.7)		*66.6 (58.0–84.0)*		*67.2 (56.2–79.0)*	

*Abbreviations: pT* histologic tumoral invasion, *Nstatus* lymph node status, *F-up* follow-up, lymph node ratio (Number of positive lymph nodes/Total number of lymph nodes)

^a^median (min-max) were reported for lymphocyte count due to their skewed distribution

^b^CI denotes confidence interval

### Independent prognostic factors of OS

The association of pre-operative, tumoral and therapeutic post-operative factors, as well as lymphocyte counts with OS in univariate analysis is shown in Table 2. We identified 9 variables as prognostic factors for OS in the univariate analysis: age at surgery (*p* = 0.003), serum albumin (*p* = 0.009), lymph nodes ratio ≥ 0.2 (*p* < 0.0001), histological grade (*p* = 0.007), venous emboli (VE) (*p* = 0.004), adjuvant chemotherapy (ACT) (*p* < 0.0001), pre and post-operative lymphocyte counts (*p* = 0.0023 and *p* = 0.0065 respectively).

**Table 2:** Univariate analysis of pre-operative, tumoral post-operative, therapeutic post operative and lymphopenia parameters for association with Overall Survival (*N* = 390)

		Number of patients	Number of Deaths			
				HR	95 % CI^a^	*P*
Pre-operative parameters	Age at surgery — years					
	≤ 65	174	130	1		
	> 65	216	179	1.405	[1.120; 1.764]	0.0034
	Patient sex					
	Male	222	169	1		
	Female	168	140	1.224	[0.978; 1.533]	0.0769
	Diabetes					
	No	252	199	1		
	Yes	73	61	1.188	[0.891; 1.584]	0.2416
	Albuminemia	191	154	0.973	[0.953; 0.993]	0.0089
	Tumor Size in cm	301	233	1.011	[0.931; 1.099]	0.7899
Post-operative tumoral parameters	pT Local invasion					
	0-1-2	39	27	1		
	3–4	336	270	1.331	[0.895; 1.980]	0.1575
	N status					
	0	119	89			
	1	269	218	1.148	[0.897; 1.470]	0.2740
	Lymph Nodes ratio					
	< 0.2	279	205			
	≥ 0.2	104	96	1.759	[1.373; 2.252]	<0.0001
	Positive lymp nodes ratio (N+/Total number of lymph nodes)	380	300	3.308	[1.962; 5.577]	<0.0001
	Histological Grade					
	Poorly differentiated	85	67	1		
	Moderately differentiated	158	127	0.797	[0.592; 1.072]	
	Well differentiated	88	64	0.672	[0.476; 0.947]	0.0735
	Vascular invasion					
	No	242	179	1		
	Yes	119	102	1.437	[1.125; 1.836]	0.0037
	Lymphatic invasion					
	No	230	174	1		
	Yes	134	110	1.199	[0.944; 1.524]	0.1374
	Perineural invasion					
	No	138	103	1		
	Yes	227	183	1.172	[0.920; 1.493]	0.1989
Therapeutic post-operative parameter	Adjuvant Chemotherapy*— no. (%)*					
	No	85	73	1		
	Yes	251	194	0.505	[0.385; 0.663]	<0.0001
Lymphopenia parameters	Pre-operative lymphocyte count	390	309	0.692	[0.546; 0.877]	0.0023
	Post-operative lymphocyte count	301	225	0.674	[0.508; 0.895]	0.0065
	Pre-operative lymphocyte count					
	≤ 1000	110	98	1		
	> 1000	280	211	0.693	[0.545; 0.881]	0.0028
	Post-operative lymphocyte count					
	≤ 1000	65	56	1		
	> 1000	236	169	0.485	[0.356; 0.660]	<0.0001
	Pre and post-operative lymphocyte count category					
	> 1000/>1000	182	123	1		
	≤ 1000/>1000	54	46	1.486	[1.058; 2.088]	
	> 1000/≤1000	37	30	2.213	[1.476; 3.317]	
	≤ 1000/≤1000	28	26	2.340	[1.524; 3.593]	<0.0001

*Abbreviations: HR* hazard ratio, *pT* histologic tumoral invasion, *Nstatus* lymph node status, *F-up* follow-up, *Nratio* lymph node ratio (Number of positive lymph nodes/Total number of lymph nodes

^a^CI denotes confidence interval

Separate multivariate cox-analysis in conventional factors block (pre-operative, tumoral and therapeutic post-operative) identified three factors independently associated with OS: lymph nodes ratio (HR = 1.8, 95 % CI: 1.286–2.438, *p* = 0.001), venous emboli (HR = 1.5 95 % CI: 1.126–2.042, *p* = 0.007), and adjuvant chemotherapy (HR = 0.4, 95 % CI: 0.276–0.550, *p* < 0.0001) (Additional file 2: Table S1A). Similarly, a separate multivariate cox-analysis in lymphocyte count parameters identified pre and post-operative lymphocyte counts as factors independently associated with OS (Additional file 2: Table S1B; *p* = 0.02 and *p* = 0.0467 respectively). Factors identified in these two previous multivariate analyses were thereafter included in a final multivariate model presented in Table 3. The final multivariate model exhibited four parameters significantly independently associated with OS with a *p* value <0.05 and one parameter borderline probably due to a lack of power: lymph nodes ratio (*p* = 0.0001), venous emboli (*p* = 0.0114), adjuvant chemotherapy (*p* = 0.0014), pre and post-operative lymphocyte counts (*p* = 0.0128 and *p* = 0.0764 respectively). When considered as continuous pre and post-operative lymphocyte count variables, their non-parametric Spearman correlation coefficient is equal to 0.36446 (*p*-value < 0.0001). Then, there is a moderate correlation between the two parameters allowing their consideration in the final multivariate model development.

**Table 3:** Multivariate final model with Pre-operative, tumoral post-operative, therapeutic post operative and lymphopenia parameters for the association with Overall Survival (*N* = 241)

	Number of patients	Number of Deaths				
			HR	95 % CI^a^	*P*	95 % bootstrap BCA
Lymph Nodes Ratio						
< 0.2	189	132	1			
≥ 0.2	52	47	1.963	[1.385; 2.782]	0.0001	[1.37650; 2.70673]
Vascular invasion						
No	165	117	1			
Yes	76	62	1.515	[1.098; 2.089]	0.0114	[1.08206; 2.04072]
Adjuvant Chemotherapy*— no. (%)*						
No	48	38	1			
Yes	193	141	0.546	[0.377; 0.790]	0.0014	[0.34875; 0.86607]
Pre-operative lymphocyte count	241	179	0.639	[0.450; 0.909]	0.0128	[0.46779; 0.95517]
Post-operative lymphocyte count	241	179	0.731	[0.517; 1.034]	0.0764	[0.50567; 1.00832]

*Abbreviations: HR* hazard ratio, Lymph Nodes ratio (Number of positive lymph nodes/Total number of lymph nodes)

^a^CI denotes confidence interval

### Final multivariate model performance assessment

Accuracy of the model was checked regarding two parameters: discrimination and calibration, which measure the ability to separate patients with different prognosis and to provide unbiased survival predictions in groups of similar patients, respectively. Our final multivariable Cox model exhibited good calibration as shown in the calibration plot (Additional file 3: Figure S2) and acceptable discrimination (C-statistic 0.64; 95 % CI: 0.60–0.69).

With the replicated datasets (*n* = 1000) derived from the bootstrap sample procedure, uncertainties around hazard ratio estimates can be measured. Bootstrapping results for the internal validation reflect the robustness of the final model as presented in Table 3. A sensitivity analysis was performed to validate the robustness of our final model with a stratified approach. By forcing prognostic factors not involved in the multivariate analysis (T, N and age) results remained similar reflecting the robustness of our final model (Additional file 4: Table S2).

### Additional value of pre and post-operative lymphocyte count parameters for OS prediction

**Fig. 1:**
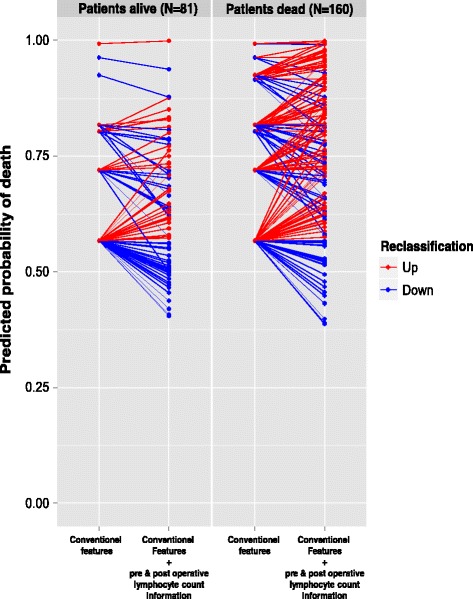
Additive value of the pre and post-operative lymphocyte count information for the reclassification of risk of death (continuous NRI) at 48 months after the diagnosis. *Blue* lines in patients without death indicate that pre and post-operative lymphocyte count information moved risk prediction in the correct (downward) direction (47/81 = 58 %). Conversely, *red* lines in patients with death indicate a correct, upward, change in risk assessment when using the pre and post-operative lymphocyte count information (94/160 = 59 %)

The inclusion of pre and post-operative lymphocyte count parameters in the reference model (including only conventional parameters) was found to significantly improve the discriminative ability of the model, because the C statistic increased significantly from 0.60–0.64 (bootstrap mean difference = 0.04; 95 % CI, 0.01–0.06). These results show that the addition of lymphocyte count parameter to clinical conventional parameters improved the stratification of patients at risk for death and then the model discrimination capacity. Similarly, the addition of lymphocyte count block to the conventional parameter block adequately reclassified at 48 months patients at lower risk for death and those at higher risk, as shown by a continuous net reclassification improvement of 0.3355 (95 % CI, 0.0719–0.5991; *p* = 0.01261; Fig. 1) and the integrated discrimination improvement was 0.03 (95 % CI, 0.01–0.06; *p* = 0.00339).

**Fig. 2:**
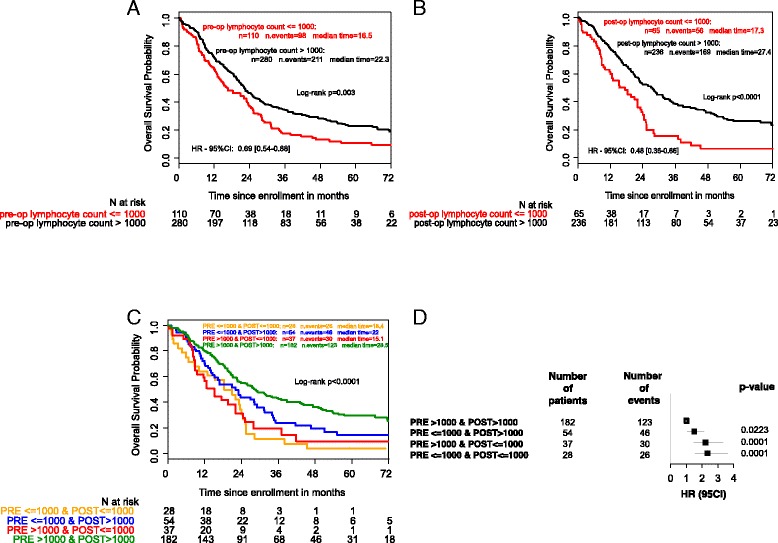
Kaplan-Meier curves for patient’s survival according to pre-operative lymphocyte count value (Panel **a**), to post-operative lymphocyte count value (Panel **b**) and to combination of the categorical (>1000; ≤1000) information in post and pre lymphocyte counts (Panel **c**). Panel **d** represent Hazard ratio for death-risk according to post and pre lymphocyte counts

Medians (minimal-maximal) of pre and post-operative lymphocyte counts in our population were 1320 (150–8350) and 1410 (200–32000), respectively. Thus, the total lymphocyte count was categorized using a threshold of 1000 cells/mm^3^. Among the 390 patients involved in the final analysis, 110 (28 %) had lymphocyte count below 1000/mm3 at baseline and exhibited different prognostic profiles for OS (*p* = 0.0028). Post-operative lymphocyte count parameter was available for 301 patients. 65 (22 %) of them had post-operative lymphopenia. A pejorative correlation with OS was also evidenced (Table 2, Fig. 2a and b, *p* < 0.0001).

By combining the categorical (>1000; ≤1000) information in pre and post-operative lymphocyte counts we identified 4 groups of patients with different prognostic profiles. Within patient’s pre and post-operative data available, 219 had a baseline lymphocyte count above 1000/mm^3^. 37 (17 %) of them were classified as lymphopenic 1 month after surgery and had poor prognosis (HR = 2.2; 95 % CI: 1.476–3.317 *p* < 0.0001). In addition, among the 83 patients of this cohort who displayed pre-operative lymphopenia, 55 (66 %) normalized their lymphocyte count after surgery and had a better prognosis (HR = 1.5; 95 % CI: 1.058; 2.088 *p* < 0.0001). Patients with the worst outcomes following surgery were those with pre and post-operative lymphopenia (HR = 2.340; 95 % CI: 1.524–3.593; *p* < 0.0001). Finally, we observed that the existence of post-operative lymphopenia categorizes patients into one group with poor prognosis (HR more than 2) whatever the pre-operative lymphocyte count. (Table 2, Fig. 2c and d).

### Long-term survivor patient’s description

Among the 390 patients enrolled, 67 (17 %) and 28 (7 %) were alive at 48 and 72 months respectively (Table 4). Lymph node ratio, venous emboli and adjuvant chemotherapy were identified as predictive factors for long-term survival.

**Table 4:** Description of the parameters of interest in the Overall population and in the long-term survivor population (>48 months and >72 months)

	Patients Characteristics	Overall population (*N* =390)		Long-term survivor (>48 months) (*N* =67)		Long-term survivor (>72 months) (*N* =28)	
		*N*		*N*		*N*	
Conventional parameters	Lymph Nodes ratio	380		67		28	
	< 0.2		279 (73 %)		59 (88 %)		27 (96 %)
	≥ 0.2		104 (27 %)		8 (12 %)		1 (4 %)
	Vascular invasion	361		65		28	
	No		242 (67 %)		51 (78 %)		23 (82 %)
	Yes		119 (33 %)		14 (22 %)		5 (18 %)
	Adjuvant Chemotherapy*— no. (%)*	336		58		22	
	No		85 (25 %)		6 (10 %)		3 (14 %)
	Yes		251 (75 %)		52 (90 %)		19 (86 %)
Lymphopenia parameters	Pre-operative lymphocyte count	390	1320 (150–8350)	67	1330 (390–3360)	28	1350 (560–2970)
	Post-operative lymphocyte count	301	1410 (200–32000)	57	1600 (930–3620)	24	1589 (940–2976)
	Pre-operative lymphocyte count	390		67		28	
	≤ 1000		110 (28 %)		11 (16 %)		6 (21 %)
	> 1000		280 (72 %)		56 (84 %)		22 (79 %)
	Post-operative lymphocyte count	299		57		24	
	≤ 1000		65 (22 %)		3 (5 %)		1 (4 %)
	> 1000		236 (78 %)		54 (95 %)		23 (96 %)
	Lymphocyte count pre and post combined	301		57		24	
	> 1000/>1000		182 (60 %)		46 (81 %)		18 (75 %)
	≤ 1000/>1000		54 (18 %)		8 (14 %)		5 (21 %)
	> 1000/≤1000		37 (12 %)		2 (4 %)		1 (4 %)
	≤ 1000/≤1000		28 (10 %)		1 (2 %)		0 (0 %)

*Abbreviations: Lymph Nodes Ratio* (Number of positive lymph nodes/Total number of lymph nodes)

Among the 67 patients alive at 48 months, pre and post-operative lymphocyte counts were available for 67 and 57 patients respectively. 56 (84 %) and 54 (95 %) patients alive at 48 months had pre and post-operative lymphocyte counts above 1000/mm3 respectively. 82 (27,2 %) patients of the overall population displayed baseline lymphopenia. Interestingly, patients who recovered an absolute lymphocyte count above 1000/mm^3^ 1 month after surgery (*n* = 54) had a similar probability to be alive at 6 years compared to the 182 patients who were never classified as lymphopenic (6 year survival rate of 10 % and 9.75 % respectively). Conversely, the 6-year survival rates of patients with lymphopenia before/after surgery or who became lymphopenic post-surgery were respectively 0 and 2.7 %. Altogether, the analysis of patients in long-term remission showed that 54 (22.8 %) and 23–236 (9.75 %) with a 1-month lymphocyte count above 1000/mm^3^ were alive at 4 and 6 years after surgery, respectively. By contrast, the probability of survival at 4 and 6 year was only 4.6 % (*n* = 3) and 1.5 % (*n* = 1) among patients with post-operative lymphopenia compared to 11 (*n* = 9) and 6 % (*n* = 5) for patients with pre-operative lymphopenia (Table 4).

## Discussion

The prognostic value of lymphopenia has been previously identified in different malignancies including pancreatic cancers [15–19, 27]. However, these results did not sustain the use of lymphopenia as a stratification criterion for clinical trials or to predict overall survival. Our study contributes to better determine the added value of lymphopenia in localized pancreatic cancer.

Neutrophil-Lymphocyte ratio (NLR) was previously proposed as an independent prognostic factor for overall survival both in localized [18–20] and in metastatic ductal pancreatic adenocarcinoma [28]. Nevertheless, NLR has some limitations. It includes two potentially independent biological factors. While neutrophils are related to inflammation, lymphocytes are directly involved in immune regulation. Moreover, NLR cut off differs from one study to another. In addition, other biological parameters related to inflammation, such as C-reactive protein, were shown to be significantly correlated to a poor clinical outcome [29].

We recorded 9 studies addressing the potential prognostic impact of pre-operative lymphopenia in localized pancreatic cancers (Additional file 5: Table S3). Only three of them identified NLR as an independent prognostic factor in multivariate analysis [18, 19, 30]. Our study confirms with statistical robustness that pre-operative lymphocyte count is an independent prognostic factor in pancreatic cancer on a larger scale (Table 3 and Fig. 2a; HR of 0,64; 95 % CI 0.450–0.909; *p* = 0.0013). The median of lymphocyte count in our cohort is in line with those observed previously in the studies of Garcea et al., and Stotz et al. [18, 31].

Having observed that 66 % of the patients in the pre-operative lymphopenia group had a total lymphocyte count above 1000/mm^3^ 1 month after surgery, the prognostic value of post-operative lymphopenia was also investigated and demonstrated. (Table 3 and Fig. 2b; HR of 0.731 (95 % CI: 0.52–1.034; *p* = 0.076). The statistical significance of the postoperative lymphopenia is supported by the good discrimination of the final model (Additional file 3: Figure S2, C-statistic 0.64; 95 % CI: 0.60–0.69), as well as the calibration analyses (bootstrap mean difference of 0.04; 95 % CI, 0.01–0.06).

Romano et al., have shown that post-operative immunodepression was significantly higher in pancreatic cancers than in colorectal and gastric cancers. Interestingly, recovery of normal post-operative lymphocyte count was longer in pancreatic cancers [32]. Only one small-scale study reported a negative relation between post-operative day 1 lymphopenia and overall survival for 111 patients with pancreatic cancer (*p* = 0.0029) [33]. However, most of the patients recovered from their decreased lymphocyte count several days following surgery and we postulated that lymphopenia monitored 1-month after the surgery might be more relevant to explore its influence on long term survival.

Of note, there are some limitations in our study. First, there are some differences between the two cohorts. Despite these differences the survival prognosis of the patients in the two cohorts was not significantly different (Additional file 1: Figure S1, Log-rank *p* value = 0.2236). From a statistical point of view, the assessment of model performance measures such as discrimination, calibration and internal validation strengthen the present investigation.

Addition of lymphocyte count variable to the conventional parameter block, in multivariate analysis, significantly improved the model discrimination capacity because the C statistic increased significantly from 0.60 to 0.64 (bootstrap mean difference = 0.04; 95 % CI, 0.01–0.06) demonstrating the additive value of lymphopenia to other conventional parameters. The use of a threshold offered better discrimination than the use of lymphocyte count because it allows death-risk stratification. In addition, combining the categorical (≤1000; >1000) information in pre and post-operative lymphocyte counts permitted the identification of several subgroups of patients with different prognoses. Patients with the worse prognosis were those with pre and post-operative lymphopenia (HR = 2.340; 95 % CI: 1.524–3.593; *p* < 0.0001). Patients who displayed lymphopenia prior to surgery and recovered absolute lymphocyte count above 1000/mm^3^ 1 month following pancreatic cancer removal had a better prognostic than patients without correction of lymphopenia (*p* < 0.0001). Consequently, the measure of lymphopenia seems more discriminant in the post-operative setting and has improved additive value for death risk stratification.

Finally, the analysis of long-term survival patients showed that 23 of the 24 patients alive 6 years after the surgery had 1-month lymphocyte count above 1000/mm^3^.

These results suggest that lymphopenia is one of the most important prognostic factors to predict long term overall survival. The impact of lymphopenia on long-term survival was also reported in metastatic patients. Indeed, a recent study including patients treated with nabpaclitaxel and gemcitabine or with gemcitabine alone reported that the number of patients alive at least 24 months after treatment initiation was increased if NLR was below 5 [28]. Such results support the inclusion of lymphopenia as a risk stratification criterion in clinical trials and in models to determine the probability of overall survival.

Prospective immunological monitoring of those patients is needed to better explain the precise mechanisms involved in lymphocyte homeostasis in pancreatic cancer patients. The role of the immune system was pointed out by studies investigating the influence of TIL on pancreatic cancer prognosis. The frequency of CD8^+^ T lymphocytes was correlated to favourable clinical outcomes and prolonged survival [34–36]. The polarization of CD4+ T lymphocytes is another possible relevant immunological parameter correlated to patients’ survival in several cancers [37]. GATA3/Tbet ratio and TH2 infiltrates were proposed as an independent prognostic factor for pancreatic cancer patients treated by surgery [38]. In this study, a predominant TH2 infiltrate was observed in peritumoral stroma suggesting a skewing of local immune responses toward TH2 polarization [38, 39]. Moreover, regulatory T cell infiltration in pancreatic cancer tissue increases during disease progression and was evidenced as a prognostic factor in resected pancreatic cancers [40]. On the other hand, lymphopenia might reflect a global metabolism alteration such as malnutrition [41]. Albuminemia was monitored in 191 out of the 390 (49 %) patients included in our cohort. We observed an influence of hypoalbuminemia on OS in univariate analysis (HR = 0.97, *p* = 0.009). Albuminemia was initially droped out of multivariate analysis due to it’s high rate of missing data. When performing the multivariate analysis with albuminemia (*n* = 152) similar results were obtained for veinous emboli and chemotherapy parameter (*p* = 0.0035 and *p* = 0.0010, respectively), even if the low number of data prompted further investigations to conclude.

However, the direct role of albuminemia as an independent prognostic factor remained unclarified, as this parameter is mostly not significant in multivariate analysis [6]. The hypothesis of a direct influence of the tumour on lymphocyte homeostasis is plausible and supported by the capacity of the tumour to secret immunosuppressive cytokines like IL-10 and TGFβ [37]. Another possible hypothesis might be the production of lymphocyte-apoptosis inducers such as Fas-Ligand, by pancreatic ductal adenocarcinoma [42].

One-month post-operative lymphopenia has independent and additive values for death risk stratification in localized pancreatic adenocarcinoma. The clinical significance of lymphopenia after surgery is highlighted by its negative correlation with the probability of long-term survival. The development of strategies based on immunonutrition [43], recombinant IL-7 [44], to expand CD4 T cells and the preliminary results of novel immunotherapies [45], offer new therapeutic endpoints to be assessed in pancreatic cancer patients.

## Conclusions

Our study demonstrated the additive value of post-operative lymphopenia to stratify pancreatic cancer patients' risk of death. Post-operative lymphopenia is an independent predictive factor of long term survival.

## Additional files

Additional file 1: Figure S1. Overall survival according to cohort set. (PDF 85 kb)
Additional file 2: Table S1. Separate multivariate cox-analysis. (PDF 290 kb)
Additional file 3: Figure S2. Calibration plots at 48 months for the final multivariate model. Vertical axis is the observed proportion of patients surviving at time of interest. Grey line represents a perfectly calibrated model. Solid line is current prediction model performance with 95 % confidence intervals using bootstrap resampling procedure. (PDF 146 kb)
Additional file 4: Table S2. Sensitivity analysis by forcing usual prognostic factors (T, N and Age) in the multivariate final model (*N* = 237). (PDF 234 kb)
Additional file 5: Table S3. Studies analysing the influence of pre-operative lymphopenia on Overall Survival. (DOCX 13 kb)

## Abbreviations

ACT: Adjuvant chemotherapy
CI: Confidence intervals
HR: Hazard Ratio
IDI: Integrated discrimination Improvement
IL-10: Interleukin-10
IL-7: Interleukin 7
NLR: Neutrophil to Lymhp
NRI: Net continuous reclassification improvement
OS: Overall survival
PDAC: Pancreatic adenocarcinoma
TGFβ: Transforming growth factor
TILs: T cell lymphocytes
