# Supplementary figures and images for: Additive value of pre-operative and one-month post-operative lymphocyte count for death-risk stratification in patients with resectable pancreatic cancer: a multicentric study
Source: BMC Cancer. 2016 Oct 26;16:823. doi: 10.1186/s12885-016-2860-6 (PMC5080693; doi:10.1186/s12885-016-2860-6)

Additional file 1: Figure S1

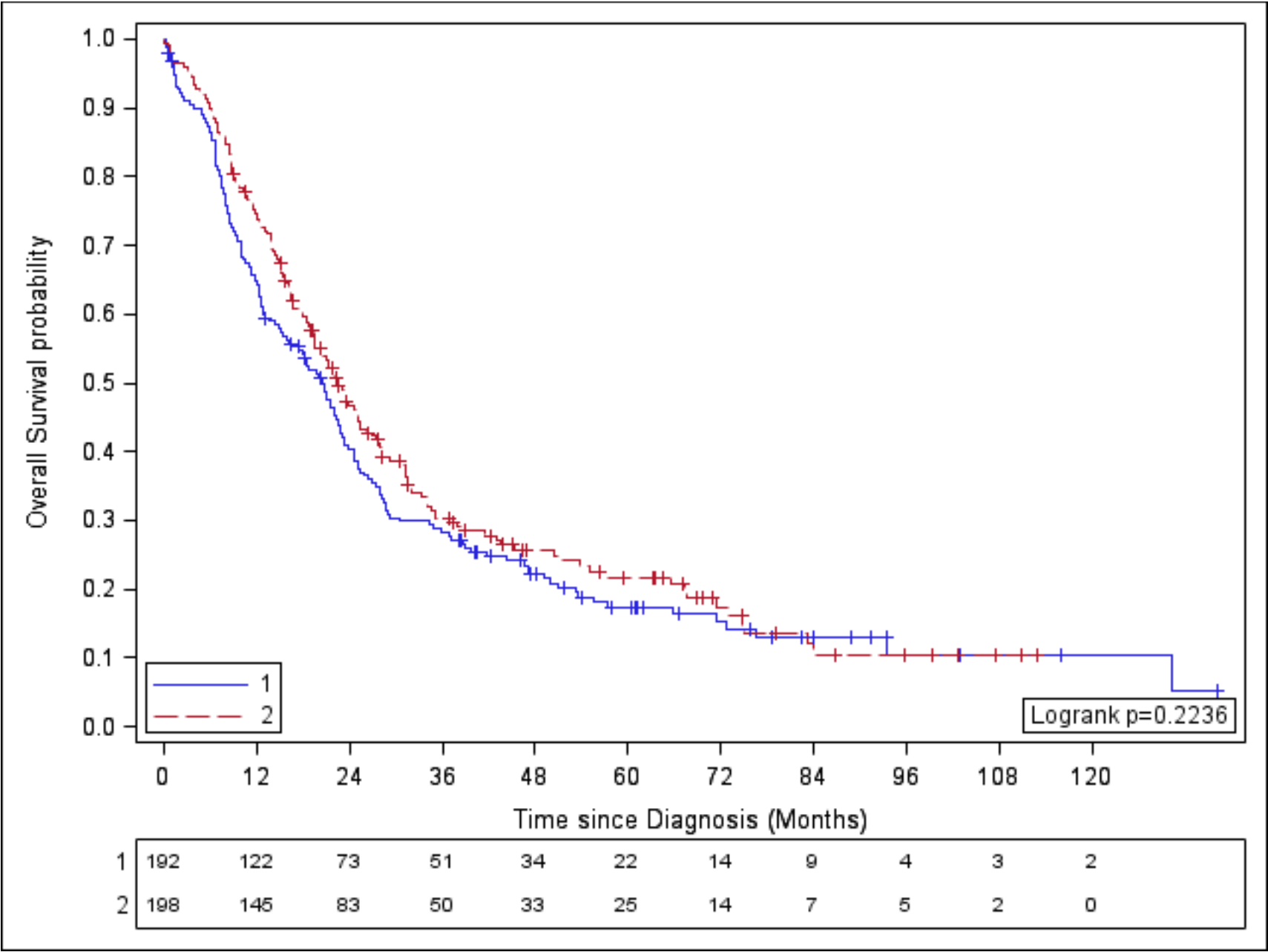

Supplement: Additional file 1: Figure S1. — Overall survival according to cohort set. (PDF 85 kb) [file 12885_2016_2860_MOESM1_ESM.pdf]

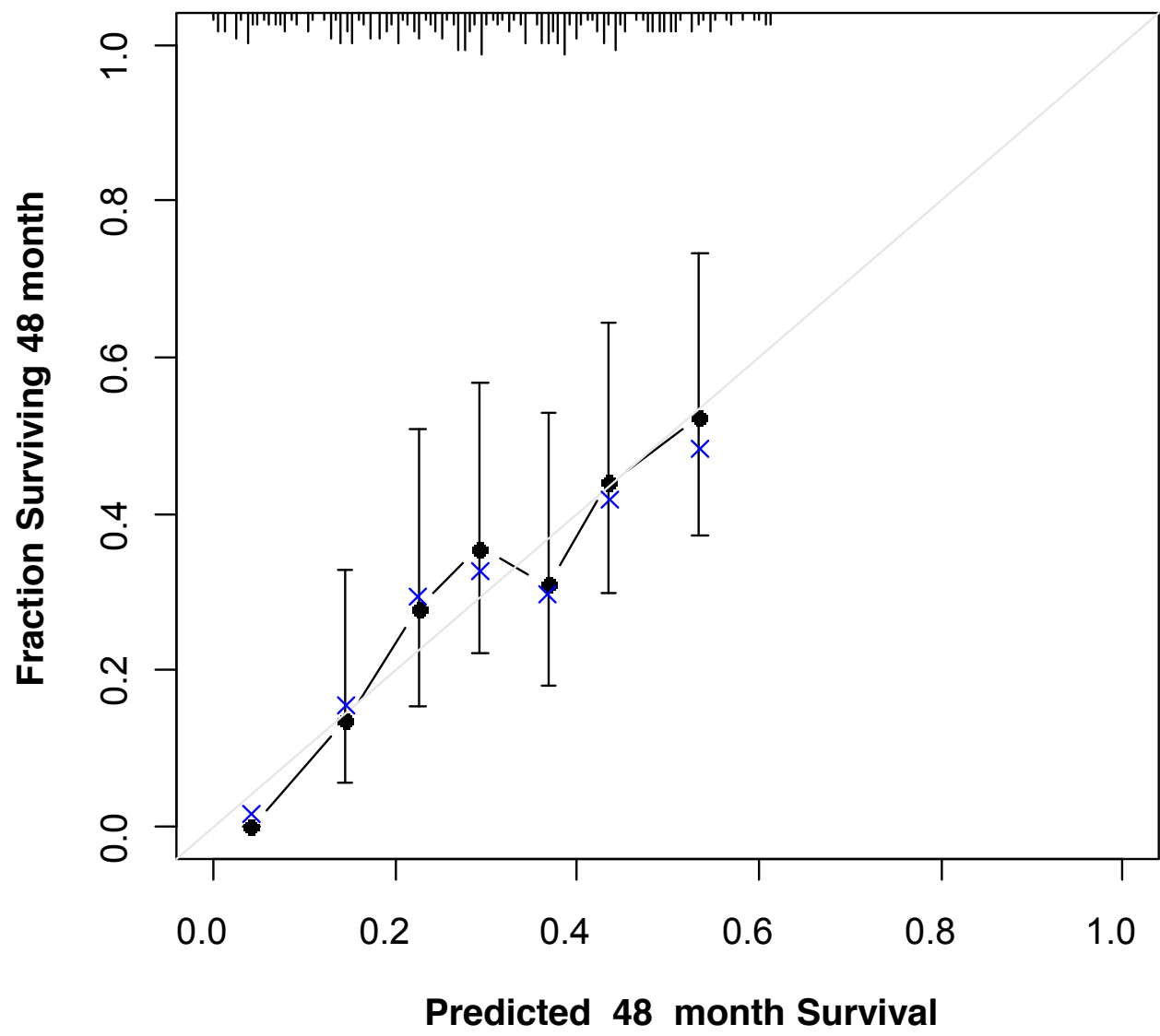

n=241 d=179 p=5, 33 subjects per group X - resampling optimism added, B=40  
Gray: ideal Based on observed-predicted

Supplement: Additional file 3: Figure S2. — Calibration plots at 48 months for the final multivariate model. Vertical axis is the observed proportion of patients surviving at time of interest. Grey line represents a perfectly calibrated model. Solid line is current prediction model performance with 95 % confidence intervals using bootstrap resampling procedure. (PDF 146 kb) [file 12885_2016_2860_MOESM3_ESM.pdf]
