# Supplementary material for: Additive value of pre-operative and one-month post-operative lymphocyte count for death-risk stratification in patients with resectable pancreatic cancer: a multicentric study
Source: BMC Cancer. 2016 Oct 26;16:823. doi: 10.1186/s12885-016-2860-6 (PMC5080693; doi:10.1186/s12885-016-2860-6)
Supplement: Additional file 2: Table S1. — Separate multivariate cox-analysis. (PDF 290 kb) [file 12885_2016_2860_MOESM2_ESM.pdf]

Additional file 2: Table S1: Separate multivariate cox-analysis.

A

	Number of patients	Number of Deaths	HR	95%CI	P
**Lymph nodes ratio**					
**<0.2**	190	137	1		
**≥ 0.2**	76	69	1.771	[1.286; 2.438]	**0.0011**
**Vascular invasion**					
**No**	180	134	1		
**Yes**	86	72	1.517	[1.126; 2.042]	**0.0067**
**Adjuvant Chemotherapy—** ***no. (%)***					
**No**	60	52	1		
**Yes**	206	154	0.390	[0.276; 0.550]	**<0.0001**

B

	Number of patients	Number of Deaths	HR	95%CI	P
**Pre-operative lymphocyte count**	301	225	0.701	[0.517; 0.950]	**0.0221**
**Post-operative lymphocyte count**	301	225	0.737	[0.546; 0.996]	**0.0467**

Table A: Separate multivariate cox-analysis for OS according to conventional parameters. Table B Separate multivariate cox-analysis for OS according to lymphocyte count parameters
Footnotes

* CI denotes confidence interval.

Abbreviations: HR: Hazard ratio; Lymph node ratio (Number of positive lymph nodes/Total number of lymph nodes
