# Supplementary material for: Additive value of pre-operative and one-month post-operative lymphocyte count for death-risk stratification in patients with resectable pancreatic cancer: a multicentric study
Source: BMC Cancer. 2016 Oct 26;16:823. doi: 10.1186/s12885-016-2860-6 (PMC5080693; doi:10.1186/s12885-016-2860-6)
Supplement: Additional file 4: Table S2. — Sensitivity analysis by forcing usual prognostic factors (T, N and Age) in the multivariate final model (N = 237). (PDF 234 kb) [file 12885_2016_2860_MOESM4_ESM.pdf]

Additional file 4: Table S2: Sensitivity analysis by forcing usual prognostic factors (T, N and Age) in the multivariate final model (N=237).

	HR	95%CI	P
**Age at surgery -- years**	1.291	[0.952; 1.750]	0.1005
**pT Local invasion — *no. (%)***			
**0-1-2**	1		
**3-4**	1.505	[0.886; 2.557]	0.1304
**N status**			
**0**	1		
**1**	0.899	[0.623; 1.296]	0.5679
**Lymph nodes ratio**			
**<0.2**	1		
**≥ 0.2**	2.035	[1.395; 2.971]	0.0002
**Vascular invasion**			
**No**	1		
**Yes**	1.484	[1.073; 2.053]	0.0170
**Adjuvant Chemotherapy— *no. (%)***			
**No**	1		
**Yes**	0.570	[0.382; 0.851]	0.0060
**pre operative lymphocyte count**	0.680	[0.477; 0.971]	0.0336
**post operative lymphocyte count**	0.700	[0.492; 0.996]	0.0477

Abbreviations: pT: histologic tumoral invasion; Nstatus : lymph node status, lymph node ratio (Number of positive lymph nodes/Total number of lymph nodes)
