# Supplementary material for: Additive value of pre-operative and one-month post-operative lymphocyte count for death-risk stratification in patients with resectable pancreatic cancer: a multicentric study
Source: BMC Cancer. 2016 Oct 26;16:823. doi: 10.1186/s12885-016-2860-6 (PMC5080693; doi:10.1186/s12885-016-2860-6)
Supplement: Additional file 5: Table S3. — Studies analysing the influence of pre-operative lymphopenia on Overall Survival. (DOCX 13 kb) [file 12885_2016_2860_MOESM5_ESM.docx]

**Additional file 5: Table S3:** Studies analysing the influence of pre-operative lymphopenia on Overall Survival.

| Study | Year | No. patients | Univariate analysis | Multivariate analysis |
| --- | --- | --- | --- | --- |
| Ben Q | 2015 | 381 | P<0.001 | P=0.003 |
| Stotz M | 2013 | 110 | P=0.006 | P=0.039 |
| Hamed M | 2013 | 85 | P=0.153 | NP |
| La Torre M | 2012 | 102 | P=0.13 | NP |
| Sanjay P | 2012 | 51 | P =0.272 | NP |
| Jamieson, NB | 2011 | 135 | P=0.51 | NP |
| Garcea G | 2011 | 74 | P=0.0057 | NP |
| Bhatti I | 2010 | 84 | P=0.023 | P=0.039 |
| Clark E | 2007 | 44 | P=0.16 | NP |

Footnotes

NP : Non Performed
